# Comparison of Revision Techniques for Rod Fracture after Adult Spinal Deformity Surgery: Rod Replacement Alone or Coupled with Lateral Lumbar Interbody Fusions or Accessory Rods

**DOI:** 10.3390/jcm13206203

**Published:** 2024-10-18

**Authors:** Ki Young Lee, Jung-Hee Lee, Gil Han, Cheol-Hyun Jung, Hong Sik Park

**Affiliations:** Department of Orthopedic Surgery, Graduate School, College of Medicine, Kyung Hee University, 23 Kyungheedae-ro, Dongdaemun-gu, Seoul 02447, Republic of Korea; keyng39@hanmail.net (K.Y.L.); gilnessxiv@gmail.com (G.H.); rucky5@naver.com (C.-H.J.); maalouf@hanmail.net (H.S.P.)

**Keywords:** accessory rod, adult spinal deformity, lateral lumbar interbody fusion, pedicle subtraction osteotomy, revision surgery, rod fracture

## Abstract

**Background:** Rod fracture (RF) is the most common cause of revision in adult spinal deformity (ASD) surgery, and various treatment strategies for preventing RF are reported in the literature. This retrospective study, involving 139 ASD patients (aged ≥65 years and a minimum 2-year follow-up) who underwent long-segment fixation from T10 to sacrum with pedicle subtraction osteotomy (PSO), analyzed long-term results, including radiographical parameters and the incidence of recurrent RF (re-RF), to determine the most effective revision method for preventing RF. **Methods:** Patients were classified into three groups according to the revision method performed for RF: simple rod replacement (RR group, *n* = 17), lateral lumbar interbody fusion around the PSO site (RR + LLIF group, *n* = 8), and accessory rod insertion (RR + AR group, *n* = 22). Baseline characteristics and radiographical and clinical parameters were analyzed. **Results:** RF occurred in 47 patients (34%) at an average of 28 months following primary deformity correction. Re-RF occurred in six patients (13%) at an average of 37 months. Re-RF occurred most commonly in the RR group (*p* = 0.048). Every re-RF in the RR group occurred at the PSO site; none occurred in the RR + LLIF group, and one in the RR + AR group occurred near the L4–5. After both primary deformity correction and revision surgery, spinopelvic parameters had shown favorable results, and clinical outcomes had improved in all three groups without significant intergroup differences. **Conclusions:** Accessory rod insertion or an additional LLIF around the PSO site seems to provide greater strength and stability to the previously fused segments than a simple rod replacement, which demonstrates the need for additional support in revision surgery for RF after a PSO.

## 1. Introduction

The reported complication rates following adult spinal deformity (ASD) surgery are as high as 70% [1], with pseudarthrosis being the major reason for a revision surgery [2]. In particular, rod fracture (RF), the most common form of pseudarthrosis, may occur even when radiographical findings show a solid bone union. Accordingly, various treatment strategies for reducing the incidence of RF following surgical treatment of ASD are reported in the literature [3,4].

ASD patients who receive deformity correction are not free from the risk of RF, as it can occur when patients accidently fall down or abruptly bend over. Moreover, the pedicle subtraction osteotomy (PSO) technique itself has been reported as a risk factor of RF [3]. Many studies to date have analyzed the related risk factors [4,5], compared the procedure-related complication risks between primary and revision surgeries [6], and explored the various complications after revision surgery [7] in the setting of ASD. However, long-term follow-up studies assessing the outcomes after revision surgeries due to RF are sparse.

In general, patients with RF are strongly advised to undergo revision not only to reduce associated pain but also to prevent the potential deterioration of sagittal balance that may result from the collapse of the vertebral body at the PSO site [3]. Although a revision surgery for RF is traditionally performed through rod replacement and supplementary posterior fusion, several alternative methods have been introduced in recent years to enhance fusion above and below the osteotomy site through a minimally invasive lateral approach and to increase both the stiffness and stability of the construct by inserting accessory rods into previous instrumentation [8,9].

The current study was conducted on ASD patients who underwent primary deformity correction via PSO and subsequent revision surgery due to RF with one of the three major revision techniques: (1) simple rod replacement, (2) lateral lumbar interbody fusion (LLIF) above and below the PSO site, and (3) accessory rod insertion. This study analyzed the long-term results, including the incidence of recurrent RF (re-RF) and the radiographical parameters, for each revision procedure.

## 2. Materials and Methods

### 2.1. Study Design

This retrospective study reviewed 139 consecutive ASD patients aged ≥65 years enrolled from 2002 to 2020 with a minimum 2-year follow-up after deformity correction via PSO. The inclusion criteria were as follows:

(1) Sagittal malalignment (sagittal vertical axis [SVA] > 50 mm, pelvic incidence [PI] minus lumbar lordosis [LL] mismatch > 10°, and pelvic tilt [PT] > 25°).
(2) Long-segment fixation with the uppermost and lowermost instrumented vertebrae at the T10 and S1, respectively.
(3) Atrophy of the back musculature in the cross-section area of magnetic resonance imaging and computed tomography (CT) in the diagnosis of lumbar degenerative kyphosis (LDK) and notable clinical signs, as previously described [10].
(4) Identification of RF based on rod breakage, with a recent fusion mass fracture being observed on plain radiography and CT and confirmed by uptakes in either bone scans or bone single-photon emission CT.

The patients were classified into three groups according to the received revision procedure: simple rod replacement (RR group), rod replacement with lateral lumbar interbody fusion above and below the PSO site (RR + LLIF group), and rod replacement with accessory rod insertion (RR + AR group).

### 2.2. Surgical Method

#### 2.2.1. Simple Rod Replacement

With each patient in a prone position, the standard posterior midline approach was made to expose the implant and confirm the site of RF. Previously inserted rods were replaced bilaterally.

#### 2.2.2. Accessory Rod Replacement

After previously inserted rods were replaced bilaterally with the standard posterior midline approach, accessory rods, each bent at the upper and lower ends, were connected to the newly replaced rods with connectors [3].

#### 2.2.3. Lateral Lumbar Interbody Fusion

In the lateral decubitus position, a blunt dissection along the muscle fibers was made to reach the retroperitoneal space. Following discectomy with contralateral annular release, a polyetheretherketone cage (12°) of appropriate height and length was chosen by inserting trial cages. A mixture of demineralized bone matrix or recombinant human bone morphogenetic protein-2 (rhBMP-2) and chipped-bone allograft was used to fill in each cage, which was subsequently inserted into the disk space above and below the PSO site. Then, previously inserted rods were replaced bilaterally with the standard posterior midline approach [3].

### 2.3. Radiographic Measurements

Sagittal alignment was evaluated using lateral 14 × 36-inch full-spine radiographs obtained with the patients standing in a neutral unsupported position with “fists-on-clavicle” [11]. All digital radiographs were analyzed using validated software (Surgimap, version 2.3.2.1, Nemaris Inc., New York, NY, USA). We evaluated PI, sacral slope (SS), PT, thoracic kyphosis (TK), thoracolumbar junction (TL), LL, lumbosacral junction (LS), and SVA. Sagittal Cobb angles were measured for TK (T5–12), TL (T10–L2), LL (T12–S1), and LS (L4–S1) [9,10]. PI, PT, and SS were measured using a standing lateral radiograph of the pelvis according to methods described previously [12].

### 2.4. RF Analysis

RF occurrence, RF site (vertebral level), and RF side (unilateral vs. bilateral) were evaluated. The surgical factors (sacropelvic fixation application and the L5-S1 fusion method) were also analyzed.

### 2.5. Clinical Outcome Measurements

Clinical outcomes were assessed using Oswestry Disability Index (ODI) and Visual Analog Scale (VAS) preoperatively, postoperatively, and at last follow-up prior to the occurrence of RF. In addition, age, bone mineral density (BMD), and body mass index (BMI), were also analyzed.

### 2.6. Statistical Analysis

Statistical analyses were performed using IBM SPSS Statistics for Windows, version 20.0 (IBM Corp., Armonk, NY, USA). Continuous variables were analyzed using one-way analysis of variance (ANOVA), Welch’s robust ANOVA, Bonferroni’s method, the Tukey HSD method, and the Dunnett T3 method for variables with normal distributions, and a Kruskal–Wallis test and the Mann–Whitney method were used for variables without normal distributions. Categorical variables were assessed using chi-square and Fisher’s exact tests, as appropriate. A *p*-value of <0.05 was considered statistically significant.

## 3. Results

### 3.1. Baseline Characteristics of Patients with RF

Patients were referred to the outpatient clinic after a startling crack sound with accompanying back pain. RF occurred in 47 patients (34%) at an average of 28 months after primary deformity correction with a mean age of 69.7 years. RF occurred at the PSO site in 39 patients (83%) and at the L4–5 level in eight patients (17%). Bilateral and unilateral RF were observed in 23 and 24 patients, respectively. Thirty-three patients had a sacropelvic fixation, and 31 and 16 patients had received ALIF and PLIF, respectively, for L5-S1 interbody fusion. Each patient received one of the following revision surgery procedures: (1) simple bilateral rod replacement (*n* = 17), (2) bilateral rod replacement with LLIF around the PSO site (*n* = 8), or (3) bilateral rod replacement with accessory rod insertion (*n* = 22).

### 3.2. Characteristics of Re-RF

Table 1 presents the characteristics of patients with re-RF. Re-RF occurred in six patients (13%) at an average of 37 months (one unilateral RF and five bilateral RF). Re-RF occurred most commonly in the RR group (*p* = 0.048), being seen in five patients (29.4%) at 15, 18, 25, 36, and 96 months, postoperatively. There was no re-RF in the RR + LLIF group. Re-RF occurred in one patient in the RR + AR group at 29 months, postoperatively. Every re-RF in the RR group occurred at the PSO site, while one bilateral re-RF in the RR + AR group occurred at L4–5 level just below each junction between the distal end of AR and the primary rod. Every patient with re-RF underwent a re-revision procedure, while one asymptomatic patient with unilateral RF underwent close observation from refusal of surgical intervention.

### 3.3. Radiographic and Surgical Features of Re-RF Patients

Table 2 shows the radiographic parameters of the three groups. Although preoperative SVA was larger in the RR + AR group than those in the other groups (*p* = 0.034), patients in all groups showed severe sagittal malalignment before primary deformity surgery. After both deformity correction and revision surgery for RF, the spinopelvic parameters of all groups showed favorable results, and sagittal alignment was well maintained prior to the occurrence of re-RF without significant intergroup differences. Also, there were no significant differences between groups with respect to sacropelvic fixation application and the L5-S1 fusion method (ALIF or PLIF) (Table 1).

### 3.4. Clinical Outcomes

The VAS for back pain and radiating pain, as well as ODI, had all improved after primary deformity surgery prior to RF without significant intergroup differences (Table 3). The lack of such differences in clinical outcomes could be attributed to the fact that the patients included in this study were elderly (age ≥ 65 years) with severe baseline sagittal imbalance and both relatively high ODI and VAS scores preoperatively. Thus, along with spinopelvic harmony, the leveled horizontal gaze and normal upright posture had already been recovered through sufficient decompression and deformity correction, which enhanced their quality of life to a great extent. Additionally, patient factors, including age, BMI, and BMD, also did not significantly differ between the three groups (Table 3).

## 4. Discussion

The restoration of sagittal balance is the main goal in the surgical treatment of ASD. Among the deformity correction methods in ASD, PSO is understandably one of the most powerful methods for achieving an ideal LL correction, which is fundamental in obtaining and maintaining optimal sagittal balance [13]. Still, there remain an array of challenges stemming from not only the complexity of the procedure itself but also from the many known complications of PSO, including RF [14,15]. Accordingly, various methods to prevent RF have been reported, such as the combination of sacropelvic fixation with long segment fusion to increase construct stability via lumbosacral fusion [16] and the insertion of multiple rod constructs for proper load distribution and posterior reinforcement at the PSO site [3].

In the setting of deformity correction of ASD, however, studies analyzing the appropriate surgical methods for revision, the long-term follow-up outcomes after revision, and the incidences of re-RF are lacking. Therefore, our study was significant in that it is the first to report on long-term outcomes, with a minimum follow-up duration of 2 years, of the three different revision methods for RF—simple bilateral rod replacement, bilateral rod replacement with LLIF around the PSO site, and bilateral rod replacement with accessory rod insertion—in ASD patients who have previously received deformity correction via long-level fusion with PSO.

### 4.1. Simple Bilateral Rod Replacement

**Figure 1:**
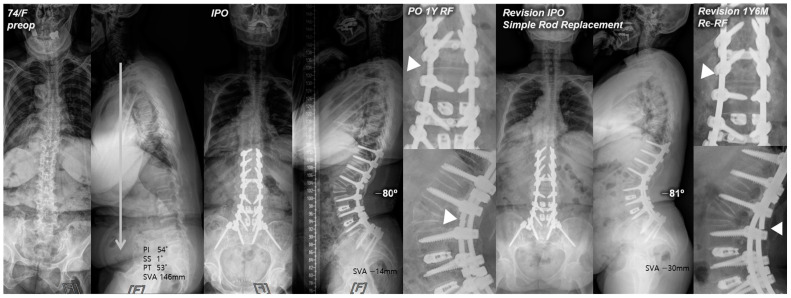
Pre- and postoperative standing radiographs of a 74-year-old female patient. After T10-S1 posterior instrumentation with PSO on L2, PLIF on L3–5, and ALIF on L5-S1, optimal sagittal balance was achieved (SVA, −14 mm; TK, 28°; LL, −80°; PI, 54°; PT, 4°; SS, 50°). At 1 year after primary deformity correction, RF (left rod) occurred at L2. At 1 year and 6 months following revision surgery with simple bilateral rod replacement, re-RF occurred at L2–3. White triangles indicate the site of RF.

Our study findings revealed the incidence of re-RF following revision surgery due to RF to be 13%. Of the three revision methods, simple bilateral rod replacement (RR group) showed the highest incidence of re-RF. We believe that the hyper-acutely contoured posterior rods paralleling a relatively large angular correction in PSO could have progressively intensified the stress concentration and lowered the fatigue strength of each rod [17,18,19,20,21], which consequently may have led to rod-breakage. Furthermore, the fact that every re-RF in the RR group occurred consistently at the same PSO site (Figure 1) not only suggests that simple bilateral rod replacement alone has a high risk of re-RF but also proves that additional support around the PSO site is ultimately required to prevent RF and maintain sagittal balance in PSO.

### 4.2. Bilateral Rod Replacement with LLIF around the PSO Site

**Figure 2:**
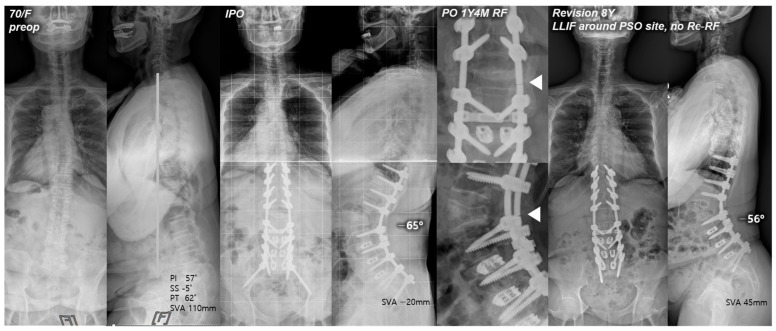
Pre- and postoperative standing radiographs of a 70-year-old female patient. After T10-S1 posterior instrumentation with PSO on L2, and PLIF on L3-S1, optimal sagittal balance was achieved (SVA, −20 mm; TK, 12°; LL, −65°; PI, 57°; PT, 17°; SS, 40°). At 1 year and 4 months after primary deformity correction, RF (right rod) occurred at L2–3. At 8 years following revision surgery with bilateral rod replacement and LLIF around the PSO site, sagittal alignment was well maintained without re-RF. White triangles indicate the site of RF.

None of the patients in the RR + LLIF group had experienced re-RF (Figure 2). This result can be attributed to the reduced residual sagittal motion of the construct, the increased stress distribution through anterior support, and the enhanced stability via interbody fusion immediately above and below the PSO site [22]. This finding was consistent with that of a cadaveric study by Deviren et al. [23] which showed increased stability through placement of interbody cages above and below the PSO site in multiaxial bending conditions. Luca et al. [8] also reported that the management of revision surgery after PSO may require an addition of anterior column support to maintain correction and reduce complications. In the same vein, Dickson et al. [24] recommended interbody fusion above and below the PSO site to help reduce the risk of further pseudarthrosis. Therefore, providing the anterior column support through interbody work around the PSO site by either a lateral or anterior approach may be a promising method for revision due to RF. However, further comparative studies are needed to assess the effectiveness of the LLIF technique with respect to the prevention of RF and postoperative complications.

### 4.3. Bilateral Rod Replacement with Accessory Rod Insertion

**Figure 3:**
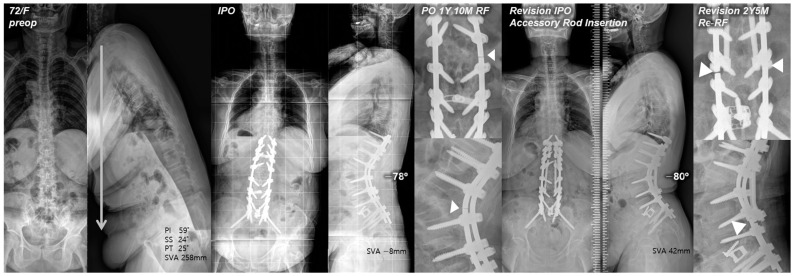
Pre- and postoperative standing radiographs of a 72-year-old female patient. After T10-S1 posterior instrumentation with PSO on L2, and ALIF on L5-S1, optimal sagittal balance was achieved (SVA, −8 mm; TK, 27°; LL, −78°; PI, 60°; PT, 12°; SS, 48°). At 1 year and 10 months after primary deformity correction, RF (right rod) occurred at L2–3. At 2 years and 5 months following revision surgery with bilateral rod replacement and accessory rod insertion, re-RF occurred at L4–5. White triangles indicate sites of RF.

Posterior reinforcement at the PSO site with multiple-rod fixation for appropriate load distribution is a crucial preventive method for RF. Numerous finite element models have demonstrated the effectiveness of additional rods in reducing stress on the primary rods across the osteotomy site [25,26]. Several clinical studies also have reported that multiple-rod fixation reduced the occurrence of RF and increased the stability at the osteotomy site [3,9]. A biomechanical study by Scheer et al. [27] that analyzed revision strategies for RF in PSO reported that multiple-rod fixation could restore stiffness and prevent fatigue in revision constructs. Therefore, multiple-rod fixation should offer proven biomechanical stability in terms of revision for RF. However, RF can still occur even with reinforcements. In our study, re-RF occurred in one of 22 patients in the RR + AR group. Interestingly, instead of occurring at the PSO site, it occurred just below each junction between the distal end of the AR and the primary rod (Figure 3). We believe that, in the application of multiple rods, connecting the distal end of the AR to the previous instrumentation at the S1–2 area could potentially offer increased stability in conjunction with L5-S1 interbody fusion and sacropelvic fixation, and further studies to confirm this are warranted.

### 4.4. Limitations

This study has several limitations. First, several variables may exist due to its retrospective nature. Second, this study examined only the patients who underwent deformity correction via PSO and subsequent revision procedures due to RF. Therefore, the number of patients with re-RF was relatively small, and the study findings may have limited implications. However, despite its limitations, this study is the first to compare the incidence of re-RF and analyze different revision methods for RF in the setting of ASD surgery.

## 5. Conclusions

For ASD patients, various revision surgery methods are available for RF following deformity correction. Our results showed that additional LLIF around the PSO site or accessory rod insertion was superior to simple rod replacement in the prevention of re-RF. Therefore, any revision surgery for RF after deformity correction with PSO should also utilize additional support to provide greater strength and stability to the previous construct. Our findings should provide an effective guideline for revisions due to RF following long posterior spinal fusion with PSO.

## Figures and Tables

**Table 1:** Baseline characteristics of re-RF patients.

Variables	RR (*n* = 17)	RR + LLIF (*n* = 8)	RR + AR (*n* = 22)	*p*-Value
Re-RF (*n* = 6)	5/12 (29.4%)	0/8 (0%)	1/21 (4.5%)	0.048 *^1^
RF detection time (month)	38	-	29	-
RF site (level)	L2–3	-	L4–5	-
RF side	1 right 4 both	-	both	-
Sacropelvic fixation	9/8	6/2	18/4	0.182 ^1^
ALIF/PLIF	11/6	4/4	16/6	0.508 ^1^

RR, simple rod replacement; LLIF, lateral lumbar interbody fusion; AR, accessory rod; RF, rod fracture; ALIF, anterior lumbar interbody fusion; PLIF, posterior lumbar interbody fusion. * Statistically significant (*p* < 0.05). ^1^ Chi-square test.

**Table 2:** Comparison of radiographic parameters between groups †.

Variables	RR (*n* = 17)	RR + LLIF (*n* = 8)	RR + AR (*n* = 22)	*p*-Value
Sagittal vertical axis (SVA, mm)				
Pre SVA	169.9 ± 67.1	169 ± 74.5	236.4 ± 98.1	0.034 *
Post SVA	−16.5 ± 17.3	−20.8 ± 29.6	−16.4 ± 27.7	0.901
SVA correction	−186.4 ± 72	−189.8 ± 84.7	−252.7 ± 97.8	0.047 *
Post Rev SVA	16 ± 33.8	6.3 ± 25.4	13.1 ± 37.4	0.805
Last SVA	36.5 ± 27.6	24.8 ± 9.7	22.4 ± 33	0.304
Thoracic kyphosis (TK, °)				
Pre TK	−2.8 ± 12	−1 ± 13.5	10.9 ± 37.8	0.407
Post TK	18.2 ± 15.1	22.6 ± 9.6	27.5 ± 10.1	0.069
Post Rev TK	32.1 ± 11.7	27.6 ± 13	35.6 ± 12.1	0.267
Last TK	31.9 ± 12	31.9 ± 13.3	39.7 ± 14.4	0.150
Thoracolumbar junctional angle (TL, °)				
Pre TL	7.5 ± 18.1	1.4 ± 17.2	11.2 ± 16.6	0.389
Post TL	−22.3 ± 19.1	−11.8 ± 23.1	−25.4 ± 16.1	0.345
Post Rev TL	−17.8 ± 22.2	−18.4 ± 16.9	−21.8 ± 9	0.971
Last TL	−17.4 ± 19.5	−15.4 ± 16.7	−20.4 ± 11.9	0.697
Lumbar lordosis (LL, °)				
Pre LL	7.6 ± 16.3	7.5 ± 14.5	11.2 ± 17.5	0.988
Post LL	−66.6 ± 16	−62.4 ± 7.4	−77.7 ± 24	0.093
LL correction	−74.2 ± 19.4	−70 ± 17.6	−88.9 ± 26.4	0.108
Post Rev LL	−61.6 ± 16.1	−62.6 ± 7.8	−70.4 ± 9.5	0.065
Last LL	−59 ± 23.5	−53.3 ± 25.5	−65.3 ± 18.6	0.376
Lumbosacral junctional angle (LS, °)				
Pre LS	−5.6 ± 19.1	0.4 ± 12.7	2.4 ± 15.1	0.383
Post LS	−24.8 ± 8.8	−27.4 ± 7.4	−27.7 ± 11.2	0.746
Post Rev LS	−22.1 ± 8.8	−27 ± 7.7	−29.4 ± 9.3	0.051
Last LS	−25.6 ± 8.7	−19 ± 11.9	−27.9 ± 11.7	0.214
Pelvic incidence (°)	55.5 ± 11.2	51 ± 10.2	57.5 ± 9.8	0.326
Sacral slope (SS, °)				
Preoperative SS	17.1 ± 14.5	21 ± 12.3	21.3 ± 13.1	0.604
Postoperative SS	42.3 ± 11.8	38.4 ± 6.9	45.7 ± 8.4	0.177
Post Rev SS	39.7 ± 13.3	40.1 ± 3.9	46.4 ± 7.4	0.074
Last SS	41.7 ± 13.4	39.4 ± 7.1	43.9 ± 8	0.538
Pelvic tilt (PT, °)				
Preoperative PT	38.4 ± 15.1	30 ± 11.3	36.2 ± 11.6	0.317
Postoperative PT	16.1 ± 9.5	16.3 ± 8.3	14.4 ± 15.6	0.894
Post Rev PT	15.8 ± 12.9	11.5 ± 7.2	10.8 ± 11.4	0.386
Last PT	13.8 ± 12.4	12.3 ± 8.2	13.4 ± 10.4	0.945
PI-LL (°)				
Pre PI-LL	63.1 ± 20.9	58.5 ± 17	68.7 ± 19.1	0.783
Post PI-LL	−11.1 ± 14.5	−11.5 ± 6.8	−20.2 ± 25.4	0.428
Post Rev PI-LL	−6.1 ± 16.3	−11.7 ± 8.6	−13 ± 11.7	0.263
Last PI-LL	−5.7 ± 19	−8 ± 10.7	−9.7 ± 12.5	0.714

† Data are presented as mean ± standard deviation. RR, simple rod replacement; LLIF, lateral lumbar interbody fusion; AR, accessory rod; Pre, preoperative; Post, postoperative; Rev, revision; Last, last follow-up. * Statistically significant (*p* < 0.05).

**Table 3:** Comparison of clinical parameters between groups †.

Variables	RR (*n* = 17)	RR + LLIF (*n* = 8)	RR + AR (*n* = 22)	*p*-Value
Age (year)	68.7 ± 6.4	69.3 ± 6.3	70.7 ± 5	0.522
BMD (gm/cm^2^)	0.89 ± 0.18	1.02 ± 0.11	0.93 ± 0.16	0.184
BMD T-score (gm/cm^2^)	−1.96 ± 1.56	−0.99 ± 1.08	−1.64 ± 1.45	0.301
BMI (kg/m^2^)	24.8 ± 3.7	27.3 ± 2.8	24.7 ± 3.7	0.211
Pre ODI	37.5 ± 2.7	37.9 ± 3.5	38.2 ± 2.4	0.675
Post ODI	18.8 ± 6	17.3 ± 4.7	19.9 ± 4	0.419
Last ODI	10.2 ± 4.2	10 ± 4.8	9.6 ± 3.6	0.986
Pre LBP VAS	8.1 ± 1.3	8.4 ± 1.2	8.6 ± 0.9	0.654
Post LBP VAS	4.5 ± 2	4.1 ± 2.4	5 ± 1.7	0.537
Last LBP VAS	1.8 ± 1.5	2 ± 1.3	1.5 ± 1.2	0.582
Pre Leg VAS	7.8 ± 0.9	8.1 ± 1.4	8 ± 1.2	0.870
Post Leg VAS	1.9 ± 1	1.8 ± 0.7	1.6 ± 0.7	0.746
Last Leg VAS	0.9 ± 0.7	1.9 ± 2.1	1.8 ± 1.7	0.419

† Data are presented as mean ± standard deviation. RR, simple rod replacement; LLIF, lateral lumbar interbody fusion; AR, accessory rod; BMD, bone mineral density; BMI, body mass index; Pre, preoperative; Post, postoperative; Last, last follow-up; ODI, Oswestry disability index; VAS, visual analog scale; LBP, lower back pain.

## Data Availability

The data presented in this study are available on request from the corresponding author. The data are not publicly available, as participants of this study did not agree for their data to be shared publicly.
